# Tracking dynamic EEG connectivity in schizophrenia and bipolar disorder

**DOI:** 10.1038/s41598-025-21747-3

**Published:** 2025-10-29

**Authors:** Aaron Maturana-Candelas, Antonio J. Ibáñez-Molina, Víctor Rodríguez-González, Sergio Iglesias-Parro, Carlos Gómez, Jesús Poza

**Affiliations:** 1 Biomedical Engineering Group, E.T.S.I. de Telecomunicación, Universidad de Valladolid, 47011 Valladolid, Spain; 2 Centro de Investigación Biomédica en Red en Bioingeniería, Biomateriales y Nanomedicina (CIBER-BBN), Madrid, Spain; 3 Instituto de Investigación en Matemáticas (IMUVA), Universidad de Valladolid, 47011 Valladolid, Spain; 4 Departamento of Psicología, Universidad de Jaén, 23071 Jaén, Spain

**Keywords:** Computational neuroscience, Biomedical engineering, Disorders of consciousness

## Abstract

Psychotic syndromes, such as schizophrenia (SCZ) and bipolar disorder (BD), significantly disrupt brain electrical activity, with functional connectivity (FC) being particularly affected. However, FC is often estimated as a static measure, overlooking the brain dynamic fluctuations that naturally occur, even at rest. In this study, we investigated alterations in dynamic FC (dFC) using resting-state electroencephalographic (EEG) data from SCZ patients, BD patients, and age-matched healthy control (HC) subjects. To achieve this, the instantaneous amplitude correlation (IAC) was computed for each EEG recording within the canonical frequency bands. We then analyzed the first- to fourth-order cumulants of the average strength (aS) time series derived from the IAC matrices. Statistically significant differences were obtained between the SCZ and HC groups in aS mean (first-order cumulant) and aS skewness (third-order cumulant) at the gamma band, while the BD group reported differences against the HC group in aS mean at the delta band. Additionally, both disorders exhibited altered aS skewness in the beta band; these findings suggest disruptions in interneuronal communication, manifesting as “pathologically Gaussian” aS distributions over time. Our results highlight the potential of dFC analysis to uncover brain function anomalies that remain undetected with conventional approaches.

## Introduction

Psychosis is a clinical condition encompassing several mental diseases that disrupt the perception of reality^1^. Among them, schizophrenia (SCZ) and bipolar disorder (BD) are particularly significant, usually exerting a devastating impact on thoughts, emotions, and behavior^1^. While their clinical manifestations differ, previous work suggests common neurotransmitter disruptions, particularly associated with dopaminergic imbalance^2^. These anomalies are believed to affect brain function in the form of frequency-dependent signal modulation in specific cortical networks^3^. Indeed, growing evidence indicates both disorders involve disruptions in brain network dynamics and neural synchronization^4–7^. These alterations may affect higher cognitive functions, such as cognition and judgment, as they arise from the brain’s ability to integrate information across distinct and specialized regions^8^. For this reason, ascertaining the disruption of the brain functional networks in psychotic states is crucial to improve our understanding of these conditions from a physiological level.

Among the techniques used to study the capability of the brain to exchange information between areas, the electroencephalography (EEG) remains one of the most widely utilized^9,10^. EEG is a non-invasive technique that allows the recording of electrical potentials caused by the activation of synchronized populations of neurons located in the cortex^11^. The popularity of the EEG is due to its portability, low cost, non-invasiveness, and outstanding temporal resolution^11^. In the context of SCZ and BD, previous EEG studies have been devoted to assessing perturbations in neural transmission of information under these conditions^12,13^. These alterations are usually quantified through statistical associations between temporal sequences from different electrodes, referred to as functional connectivity (FC)^14^. FC can be computed using various metrics, such as amplitude envelope correlation^15^ or phase lag index^16^, which are based on amplitude and phase associations, respectively. The amplitude‑based approach is particularly valuable for revealing alterations in functional brain organization within specific neural populations, especially when integrated with fMRI analyses^17^. This procedure has allowed to even develop connectivity-based predictive models to evaluate reaction times in pattern recognition tasks^18^. With respect to psychosis research, previous studies have revealed distinct patterns of resting-state FC alterations in BD and SCZ, strengthening the idea that psychotic states influence neural intercommunication. Both disorders show increased power and coherence in low frequency bands, particularly theta, compared to healthy controls^19,20^. Additionally, altered connectivity in the medial prefrontal cortex, insula, and ventrolateral prefrontal cortex distinguished BD from SCZ^21^. While some studies reported no differences in default mode, frontoparietal, and salience networks between euthymic BD patients and controls, others suggest hypoconnectivity in BD patients with a history of psychosis^22^. Aberrant connectivity involving the amygdala, prefrontal cortex, and cingulate cortex was also observed in BD, potentially reflecting trait-based pathophysiology or compensatory mechanisms^22^. These findings highlight the potential of resting-state EEG as a biomarker for differentiating between BD and SCZ.

Despite the usefulness of FC to evaluate brain functional alterations, this feature is traditionally calculated over fixed-length signal segments, limiting the ability to assess dynamic trends that evolve over time. Previous works suggest the existence of fast-paced connectivity variations in the range of milliseconds, which are sensitive to mental conditions^23,24^. Therefore, increasing the temporal resolution of FC methods is crucial for capturing a comprehensive view of connectivity anomalies in specific states. Recently, a method for estimating high-resolution FC was proposed, known as instantaneous amplitude correlation (IAC)^25^. This metric estimates the amplitude-based FC for each sample of the EEG sequence. IAC allows the incorporation of the time factor into the FC calculations, resulting in what is called dynamic FC (dFC). This novel perspective transcends the typical static approach, which disregards the time-varying connectivity fluctuations across time. “Chronnectomic” studies (i.e., connectivity integrating the concept of time) have provided new insights into the importance of dynamical variations of connectivity values to describe brain function^26^. Chronnectivity has also been applied in the context of mental diseases, such as SCZ, to get deeper insights about alterations in brain flexibility^27^. Undoubtedly, the study of sample-to-sample connectivity not only favors the understanding of the dynamic alterations of brain functional activity in multiple circumstances, but it is a recent and innovative field of research. In fact, multiple recent reviews of EEG and MEG functional connectivity show that it is not a common practice, and static methods are still widely used^10,28^. Our proposal has an additional advantage: chronnectomic metrics exploit the high temporal resolution that characterizes EEG.

To characterize brain connectivity alterations associated with BD and SCZ, we propose an exploratory study that examines the statistical cumulants of whole-brain connectivity values on a per-sample basis. We chose the use of statistical cumulants since they are the simplest and most straightforward manner to ascertain alterations in the sequences describing dFC. This work extends a previous study focused on dFC in SCZ patients^29^, now including BD patients to identify shared connectivity disruptions across psychotic states over time. We hypothesize that these cumulant-based variables, which capture the temporal distribution of connectivity values, are altered in psychotic patients even during resting-state conditions. This approach may reveal novel neurophysiological features that typically remain undetected when using conventional FC analyses.

## Materials and methods

### Participants

This study involved 41 participants, divided into three groups: 20 healthy controls (HC), 10 individuals diagnosed with BD, and 11 individuals diagnosed with SCZ. The data were collected in previously published studies from our laboratory, and the methods for recruitment, data acquisition, and preprocessing have been described in detail in those publications^30–32^.

The 10 BD patients were recruited from the San Agustín University Hospital (Linares, Spain). To be included in the BD group, participants were required to have a diagnosis of bipolar disorder (F31) according to the International Classification of Diseases (ICD-10), as determined by the clinician in charge of the patients. This group comprised 5 men (50%) and 5 women, with a mean age of 40.60 ± 11.28 years (mean ± standard deviation, SD); all of whom were right handed. Educational levels varied with 3 patients having primary education, 6 having secondary education, and 1 having higher education. The average duration of the disorder was 27.23 ± 7.66 years (mean ± SD). Participants varied on medication. Most of them were on stabilizers (lithium or valproic acid), anxiolytics (flurazepam, lormetazepam or clonazepam), and antipsychotics (quetiapine, aripiprazole and paliperidone). Antipsychotics were converted to chlorpromazine equivalents, averaging 211.9 ± 192.0 mg a day (mean ± SD). The SCZ group included 11 participants recruited from the Mental Health Day Centre at San Agustín University Hospital. Participants met the inclusion criteria of having an ICD-10 diagnosis of schizophrenia (F20) and schizoaffective disorder (F25), according to a broad definition of the psychosis spectrum. This decision is based on the current conceptualization in international diagnostic systems (DSM-5, ICD-10/11), which consider schizoaffective disorder as part of the psychotic spectrum due to the presence of independent and persistent psychotic symptoms. Patients with affective disorders with psychotic symptoms occurring only during affective episodes were excluded. The average age of the SCZ group was 36.2 years (SD = 10.2), with ages ranging from 23 to 53 years. The group comprised 2 women (18%) and 9 men, all of whom were right-handed. Educational levels varied, with 2 participants having primary education, 8 having secondary education, and 1 having higher education. The average duration of the disorder was 15.7 ± 10.2 years (mean ± SD), with a range of 3 to 35 years since diagnosis. All participants were on atypical antipsychotic medication, with 2 receiving oral medication and 9 receiving injectable forms. Additionally, one participant was also on antidepressants. Antipsychotic doses were converted to chlorpromazine equivalents, averaging 818.2 ± 407.8 mg a day (mean ± SD). The inclusion of schizoaffective disorder in the SCZ group is based on evidence that its clinical, neurobiological, and cognitive characteristics lie on a continuum with SCZ, and that psychotic symptoms are the core diagnostic feature distinguishing it from affective disorders. This methodological strategy allows for comparison of EEG temporal complexity between primary psychosis, affective disorders, and healthy controls, minimizing diagnostic overlap and facilitating interpretation of the results.

Psychotic symptoms in the SCZ and BD groups were assessed using the Spanish version of the Positive and Negative Syndrome Scale (PANSS)^33,34^. The PANSS comprises 30 items rated on a 7-point scale, where 1 indicates the absence of the symptom and 7 denotes the presence of the symptom with extreme severity. This version of the PANSS includes three subscales: positive, negative and general. The positive subscale with 7 items evaluates symptoms such as delusions, conceptual disorganization, hallucinations, excitement, grandiosity, suspiciousness/persecution, and hostility; these symptoms reflect a distortion or exaggeration of normal functions. The negative subscale with 7 items measures symptoms such as affective flattening, emotional withdrawal, poor rapport, social withdrawal, reduced abstract thinking, decreased fluency of conversation, and stereotyped thinking. These symptoms indicate a diminution or loss of normal functions. The general psychopathology subscale with 16 items, which assesses the presence of other symptoms such as depression, anxiety, and disorientation. Exclusion criteria for SCZ and BD patients included a concurrent diagnosis of a different neurological disorder, substance abuse disorder, history of developmental disability, vision disorders (*e.g.*, cataracts affecting visual acuity despite correction), hearing disorders (unless corrected with hearing aids or surgery), and inability to provide informed consent. Data regarding the PANSS scoring for each group are displayed in Table 1.

On the other hand, the HC group consisted of 20 participants recruited from the University of Jaén, an adult school in Jaén, and the staff of San Agustín University Hospital. The mean age in this group was 40.7 ± 11.9 years (mean ± SD), with ages ranging from 23 to 57 years. The group included 7 women (35%) and 13 men, with 18 participants being right-handed. Educational backgrounds included 1 participant with primary education, 12 with secondary education, and 7 with higher education. Statistical tests revealed no significant differences between the groups in terms of educational level ($\chi ^{2}$(2) = 3.29, *p*-value = 0.19) or sex ($\chi ^{2}$(2) = 0.97, *p*-value = 0.32). Age comparison using the Mann-Whitney *U*-test showed no significant differences (*U*-value = 85, *p*-value = 0.31). For the HC group, an additional exclusion criterion was a self-reported diagnosis of a mental disorder. All participants provided written informed consent in accordance with the Declaration of Helsinki, and the study was approved by the Jaén Research Ethics Committee.

**Table 1:** PANSS scores attributed to BD and SCZ patients for each PANSS subscale. *SD* standard deviation; *BD* bipolar disorder; *SCZ* schizophrenia.

	Positive PANSS (mean ± SD)	Negative PANSS (mean ± SD)	General PANSS (mean ± SD)
BD group	11.77 ± 3.98	13.46 ± 5.97	27.33 ± 7.66
SCZ group	14.27 ± 5.62	20.22 ± 7.78	30.72 ± 8.14

### EEG data recording and preprocessing

Resting-state EEG data were recorded in controlled sessions where participants were comfortably seated in one of the laboratories at San Agustín University Hospital. Participants were instructed to focus on a light grey fixation cross placed in the center of a black background on a laptop screen positioned approximately 70 cm from their eyes. They were asked to remain still and think about whatever they wanted in silence. A researcher was present in the room but remained out of the participants’ view. EEG signals were recorded using a BrainAmps amplifier equipped with 31 active electrodes placed according to the standard 10-20 system. The electrode positions included FP1, FP2, F7, F3, Fz, F4, F8, FT9, FC5, FC1, FC2, FC6, FT10, T7, C3, C4, T8, TP9, CP5, CP1, CP2, CP6, TP10, P7, P3, Pz, P4, P8, O1, Oz, and O2. All electrodes were referenced to the Cz electrode, and impedances were kept below 5 k$\Omega$. The EEG signals were sampled at a frequency of 500 Hz.

EEG preprocessing was conducted using EEGLAB^35^ and custom MATLAB^®^ scripts. The preprocessing steps included the following: i) band-pass FIR (finite impulse response) filtering (order 500, Hamming window) with cutoff frequencies between 0.5 and 98 Hz to limit noise bandwidth, preserving the frequency bands of interest. ii) notch FIR filter at 50 Hz (order 500, Hamming window) to diminish the power-grid interference; iii) independent component analysis (InfoMax algorithm) to remove components related with artifacts such as blinks, eye movements, and muscle activity; iv) selection of continuous 60 seconds of clean data for each participant.

### EEG analysis

The dFC was studied for each EEG recording by means of IAC^25^, which reflects the temporal evolution of the connectivity values of the brain functional network. Technically, IAC consists in the correlation in terms of amplitude between two sequences sample by sample. In the context of EEG, IAC returns an adjacency matrix that encompasses the connectivity values between each pair of electrodes for each temporal sample. IAC between sequences *i* and *j* is defined as follows^25^:

1 $$\begin{aligned} IAC_{i,j}(t) = \hat{E}_{i}(t) \circ \hat{E}_{j}(t), \end{aligned}$$

where $\circ$ represents the Hadamard product and $\hat{E}(t)$ the normalized amplitude of the envelope. IAC allows the characterization of connectivity pattern fluctuations with higher temporal resolution than other metrics^25^. This perspective has been previously used to study the recurrence of connectivity pattern configurations under pathological states^23,27^. In order to minimize spurious correlations due to volume conduction effects, the EEG was previously orthogonalized^36^. In this study, IAC calculation resulted in an adjacency matrix for each of the 30,000 EEG temporal samples (60 s x 500 Hz) in each of the classical frequency bands for each participant. Frequency bands are defined as delta (0.5-4 Hz), theta (4-8 Hz), alpha (8-13 Hz), beta (13-30 Hz), and gamma (> 30 Hz). The code for the computation of the IAC can be found on GitHub (https://github.com/Prejaas/High-temporal-resolution-MEG-measures-of-functional-connectivity).

**Fig. 1:**
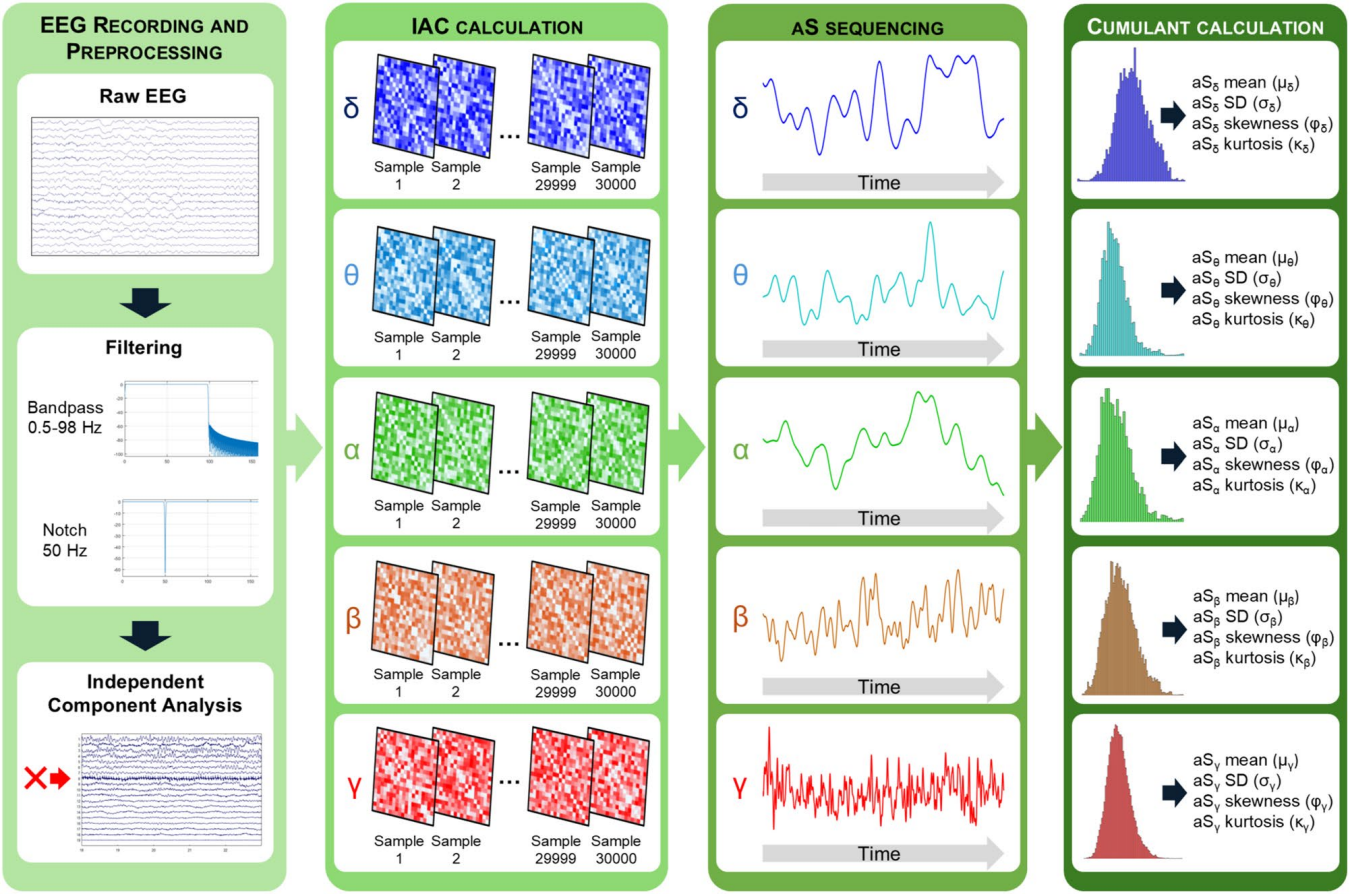
Processing pipeline used to compute each statistical cumulant from the aS sequence: The process begins with EEG being preprocessed and filtered in each classical frequency band. Subsequently, IAC is calculated for each sample in each frequency band. Then, the grand average (average strength, aS) among ROIs is calculated, resulting in a single value for each sample. Finally, the aS sequence is obtained and the cumulants are computed.

**Fig. 2:**
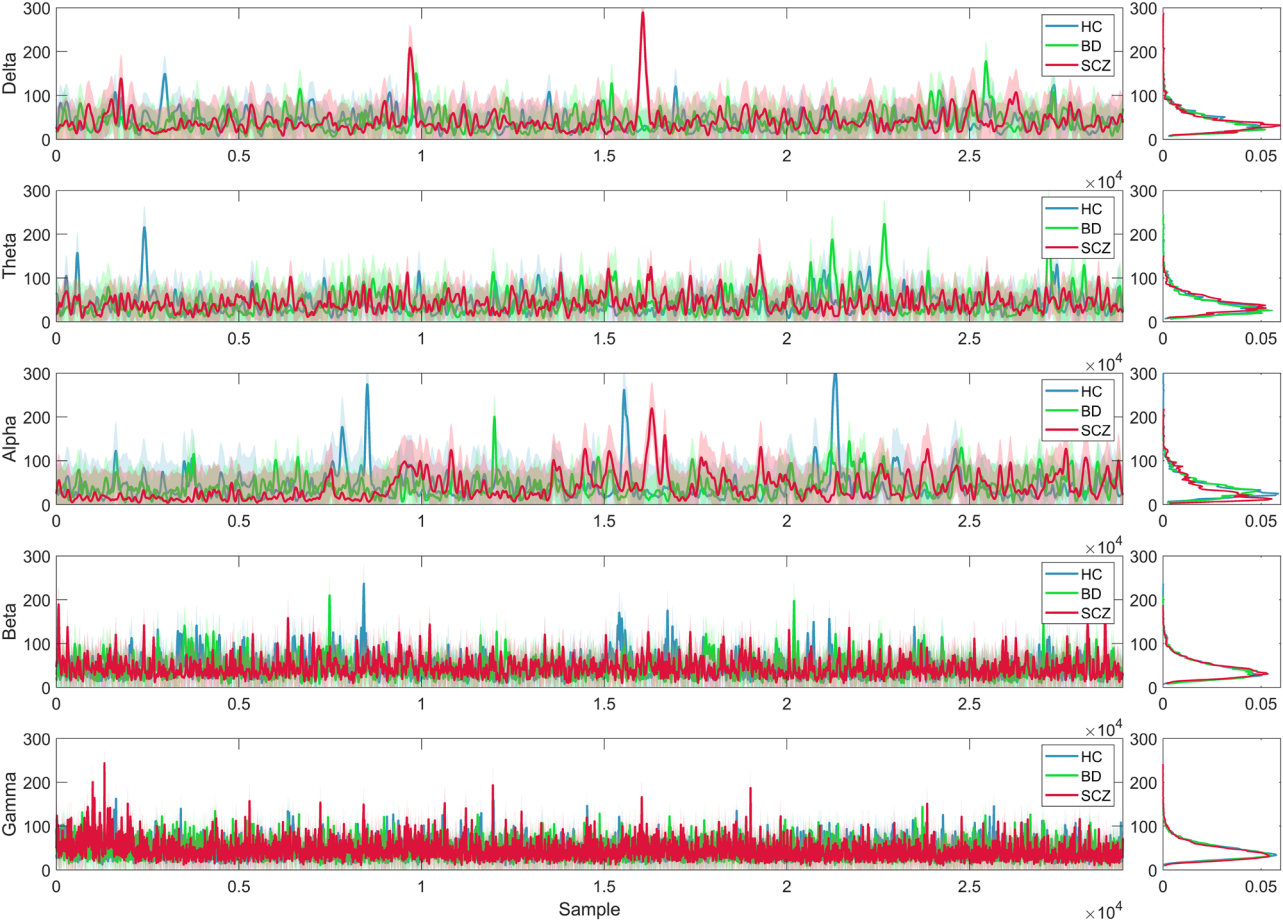
Example of aS sequences in each frequency band obtained from a random subject in each clinical group: HC: Healthy subjects in blue, BD: Bipolar disorder patients in green, SCZ: Schizophrenia patients in red. The distributions of the aS values for each subject are displayed on the right.

The time-varying global node average strength (aS) across channels for each subject was calculated from the IAC matrices. Afterwards, statistical cumulants up to fourth order (mean, $\mu$; standard deviation, $\sigma$; skewness, $\varphi$; and kurtosis, $\kappa$) were obtained to quantify different aspects of functional brain network dynamics during the resting state. While $\mu$ represents the average node strength over the 60-second signal, $\sigma$ estimates the variability of node strength values around the mean. Furthermore, $\varphi$ measures the asymmetry of the distribution, indicating whether node strength values are skewed towards higher or lower ranges. Lastly, $\kappa$ assesses the “tailedness” of the distribution, highlighting the presence of outliers and the sharpness of the distribution peak. In order to improve clarity, a scheme representing the pipeline of the method is illustrated in Figure 1. As an example, the resulting aS sequences for a single subject from each group across frequency bands are displayed in Figure 2.

## Results

### Correlation between chlorpromazine equivalency and aS metrics

The impact of psychotropic drugs on EEG readings is widely known^37^. Concretely, antipsychotic drugs for schizophrenia treatment have been associated with EEG alterations in specific frequency bands^37^. Since every SCZ patient was under drug prescription, their effects on the EEG have to be previously asserted. To this end, the Pearson correlation between each calculated parameter and the chlorpromazine equivalent was obtained for each frequency band. No *p*-values below the significance threshold ($\alpha < 0.05$) were obtained for any metric in any frequency band. Rho and *p*-values for each calculation are exhibited in Table 2.

**Table 2:** Rho and *p*-values obtained from the Pearson correlation between chlorpromazine equivalency and each parameter for each frequency band. $\mu$: mean; $\sigma$: standard deviation; $\varphi$: skewness; $\kappa$: kurtosis.

Frequency band	aS $\mu$		aS $\sigma$		aS $\varphi$		aS $\kappa$	
	Rho	*p*-value	Rho	*p*-value	Rho	*p*-value	Rho	*p*-value
Delta	-0.262	0.436	-0.024	0.944	0.001	0.976	-0.041	0.903
Theta	0.087	0.809	-0.185	0.586	0.198	0.560	0.367	0.268
Alpha	0.519	0.102	-0.082	0.812	0.007	0.984	-0.005	0.989
Beta	-0.065	0.849	-0.190	0.575	-0.111	0.747	-0.075	0.828
Gamma	-0.02	0.994	-0.242	0.474	-0.159	0.641	-0.094	0.783

### Statistical differences in aS cumulants between groups

**Fig. 3:**
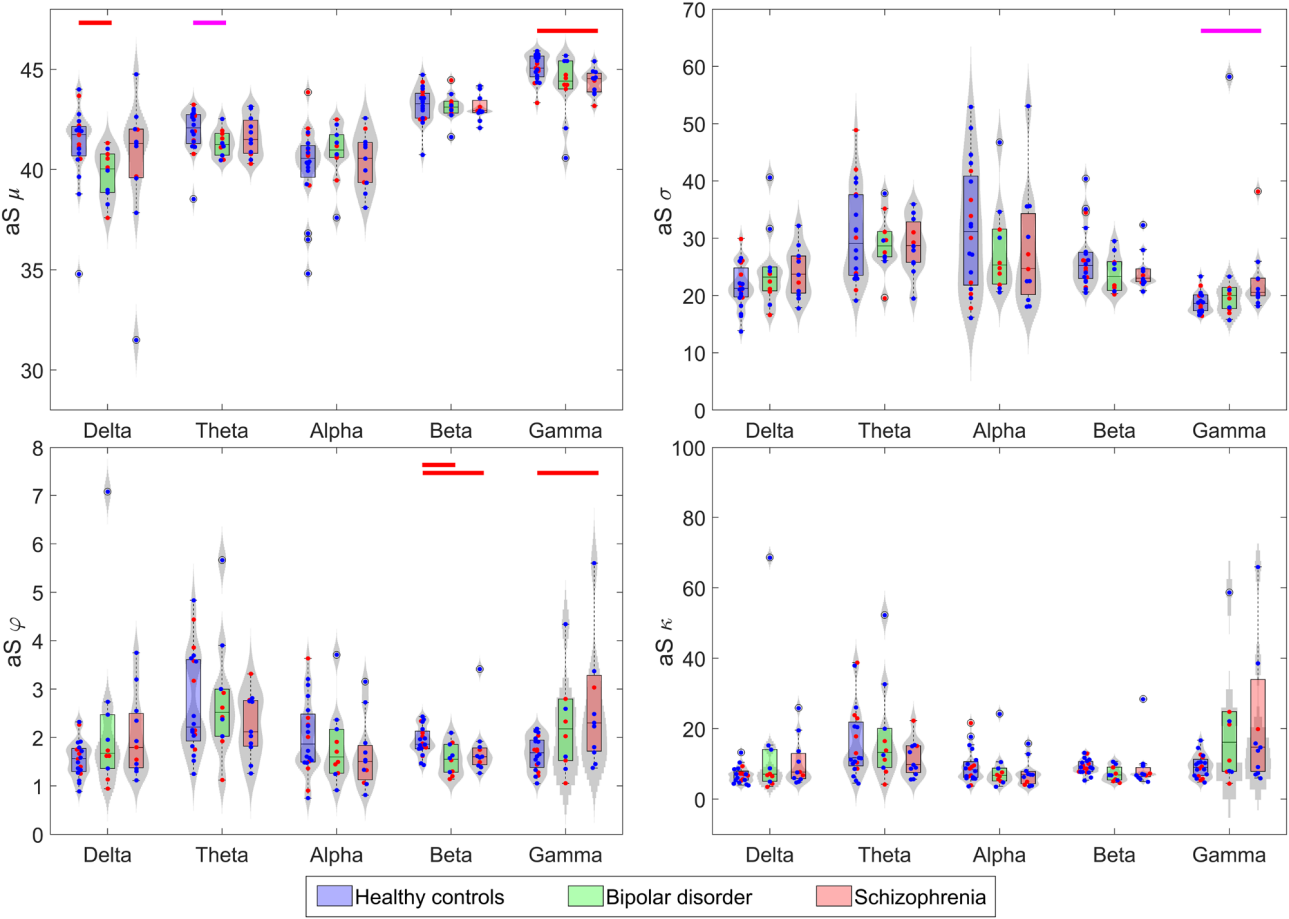
Distribution of mean ($\mu$), standard deviation ($\sigma$), skewness ($\varphi$), and kurtosis ($\kappa$) of the aS sequences for each frequency band. Blue boxes represent healthy controls, green boxes bipolar disorder patients, and red boxes schizophrenia patients. Significant differences between groups (*p*-value < 0.05, FDR-corrected Mann-Whitney *U*-test) are displayed as red dashes. Noteworthy tendencies (0.05 < *p*-value < 0.1, FDR-corrected Mann-Whitney *U*-test) are shown as magenta dashes. Values corresponding to men are shown in blue dots and values corresponding to women are shown in red dots. No significant differences were obtained in any group and metric between sexes (*p*-value > 0.05, Mann-Whitney *U*-test).

The first- to fourth-order cumulants of the aS distributions were computed, that is, aS mean ($\mu$), standard deviation ($\sigma$), skewness ($\varphi$), and kurtosis ($\kappa$). The resulting values are illustrated in Figure 3. In the case of BD patients, statistically significant differences were found regarding controls in aS $\mu$ in delta band (*p*-value = 0.044, *U*-value = 162, effect size (*r*) = 0.498, Mann-Whitney *U*-test corrected with False Discovery Rate, FDR) and aS $\varphi$ in beta band (*p*-value = 0.047, *U*-value = 157, *r* = 0.458, FDR-corrected Mann-Whitney *U*-test). Also, differences close to the statistical significance threshold were obtained in the theta band in aS $\mu$ (*p*-value = 0.098, *U*-value = 150, *r* = 0.402, FDR-corrected Mann-Whitney *U*-test). On the other hand, SCZ patients showed statistically significant differences in aS $\mu$ in gamma band (*p*-value = 0.044, *U*-value = 174, *r* = 0.475, FDR-corrected Mann-Whitney *U*-test) and aS $\varphi$ in beta (*p*-value = 0.044, *U*-value = 170, *r* = 0.444, FDR-corrected Mann-Whitney *U*-test) and gamma bands (*p*-value = 0.044, *U*-value = 46, *r* = 0.475, FDR-corrected Mann-Whitney *U*-test). Finally, gamma band aS $\sigma$ exhibited differences close to the statistical significance threshold (*p*-value = 0.053, *U*-value = 42, *r* = 0.504, FDR-corrected Mann-Whitney *U*-test).

## Discussion

In this study, the alterations in EEG-derived dFC were evaluated in BD and SCZ patients. The values of up to fourth-order cumulants of aS throughout the EEG recording exhibited distinct trends between pathological groups and healthy controls in specific frequency bands. Firstly, the aS $\mu$ values (*i.e.*, the mean of the aS sequence) showed significant differences between control subjects and BD patients in delta band. This observation was noticeable but less evident in the theta band. This indicates that connectivity values are generally higher over time in slow rhythms of brain electrical activity in healthy subjects. These results align with other studies, which reported connectivity alterations in delta and theta bands in psychotic patients across multiple conditions^5,38^, which were related to poorer connectivity performance between diverse neural populations^38^. Interestingly, hypoconnectivity in the anterior and posterior regions of the default mode network (DMN) in BD patients was identified in previous work^39,40^. Therefore, brain connectivity alterations exerted by BD could be associated with disruptions in slow frequency oscillations globally present in resting-state networks across the brain. It is noteworthy that drug administration, such as antipsychotics and benzodiazepines, caused minimal effect on connectivity values^5^. In addition, previous studies have suggested that low-frequency bands primarily facilitate synchronization between distant brain regions, whereas higher frequencies are typically associated with synchronization within local cortical networks^41,42^. Therefore, a reduction in aS $\mu$ values in BD patients could be related to a generalized cerebral disconnection in the resting-state. It is well known that the DMN is particularly active in this state and contributes to large-scale functional organization^43^. Since the DMN encompasses extensive and dispersed regions in the brain^43^, and given that hypoconnectivity between the DMN and other widespread regions has previously been demonstrated in BD patients^44^, the observed results may be a consequence of this phenomenon.

Regarding the aS $\mu$ parameter calculated from the SCZ patients, these reported lower values at the gamma band compared to the control group. Furthermore, a trend of higher aS $\sigma$ (i.e., the standard deviation of the aS sequence) was also observed in this frequency range. These alterations may reflect disruptions in the stability of networks involved in cognitive processing^45^. SCZ has been previously linked to disruptions in gamma-band activity^46,47^. The observed reduction in gamma connectivity in SCZ during the resting state could be attributed to the decreased gamma power under similar conditions, as reported in other studies^48,49^. Additionally, the relation between gamma alterations in SCZ, excessive synaptic plasticity, and abnormal cholinergic activity has been previously suggested^46^.

With respect to the aS $\varphi$ values (i.e., the skewness of the distribution of aS values), both pathologies reported a statistically significant decrease in beta band. Additionally, aS $\varphi$ values exhibited positive numbers, reflecting distributions to skew into lower values. Variations in aS $\varphi$, without affecting other cumulants, indicate changes in the Gaussian properties of the aS value distribution but not in its magnitude. These findings suggest efficiency alterations in neural connectivity within localized regions, as faster frequency bands have been linked to interactions among local neuronal populations^41^. Therefore, the observed aS $\varphi$ changes in beta could reflect disruptions in the complexity of communication between small, specialized brain regions. Notably, aS $\varphi$ in the beta band was altered in both pathological groups. Mental disorders associated with psychosis, such as SCZ and BD, have been strongly linked to neurotransmitter deficits, with dopamine imbalance being particularly prominent in these conditions^2^. Recent research has shown that the circuit architecture underlying beta activity is particularly susceptible to dopamine fluctuations^50^. Chickermane’s work suggested that beta oscillations arise within cortico-subcortical brain networks modulated by this neurotransmitter^50^. This indicates dopamine anomalies could be linked not to dFC values on average, but rather to their distribution over time. From our results, the aS $\varphi$ values are lower for both pathologies than in the control group, being closer to zero. This means that pathological states are associated with more Gaussian aS fluctuations. While counterintuitive at first glance, given the common association of psychosis with neural “chaos”, this statistical normalization may reflect a critical loss of adaptive complexity. Healthy neural systems exhibit leptokurtic distributions (heavy-tailed) that enable rapid transitions between metastable states, a hallmark of cognitive flexibility^51^. The observed Gaussianization in BD and SCZ aligns with spectral entropy studies showing reduced dynamic repertoire exploration^27^, suggesting a pathological rigidity in state-space navigation. Previously, psychedelic states (characterized by distorted perception of reality) have been associated with a greater diversity of connectivity motifs^52^. This increase in the dynamical repertoire^52^ could influence the aS $\varphi$ parameter, making it more Gaussian. In addition, more normalized distributions in beta dFC could suggest a reduction in the presence of states of high functional synchronization, something that could explain deficits in executive functions and cognitive control in BD and SCZ. Clinically, the Gaussianity index emerges as a promising transdiagnostic biomarker of cognitive rigidity, particularly valuable given its independence from antipsychotic dosage in our cohort.

Finally, the SCZ group exhibited extended aS $\varphi$ alterations into the gamma band. In this case, changes in aS $\varphi$ were accompanied by modifications in the aS $\mu$ parameter, possibly reflecting more pronounced gamma-band disruptions characterized by abnormal clustering of oscillations within narrower frequency ranges. In a previous study, decreased EEG predictability was reported in both the time and frequency domains in SCZ patients^53^. In line with our findings, the increased entropy observed by Sabeti et al.^53^ may stem from higher variability in faster oscillations. This phenomenon has been associated with disturbances in the small-world properties of functional brain networks in SCZ^53^. From a physiological perspective, impairments in GABAergic neuronal circuitry^47^ may disrupt the ability to maintain gamma network oscillations, potentially compounding the effects of other neurotransmitter deficits. Gamma anomalies could also extend to ionotropic receptors in SCZ. In this case, the *N*-methyl-D-aspartic acid (NMDA) receptor was found to affect gamma network oscillations in the hippocampus, affecting metabolic glutamate receptors in cortical neurons^54^. Since these neuronal structures work together to generate better synchronized and distributed gamma oscillations^54^, the alterations perceived in gamma aS skewness could be a direct manifestation of this phenomenon.

Despite the valuable findings of this study, several limitations should be acknowledged. A primary constraint is the limited sample size, which affects statistical power and the generalizability of the results. Additionally, an imbalance between control subjects and patients was present for BD and SCZ patients, which could limit the statistical analyses. We also observed a sex imbalance in the SCZ group, which reflects the natural distribution of these disorders in the population^55,56^. However, we recognize that sex differences may impact clinical and neurobiological outcomes. Expanding the dataset in future studies will be crucial to strengthen the reliability of our findings. Moreover, while this study focuses on global connectivity measures, incorporating alternative connectivity metrics could provide a more comprehensive perspective on brain network dynamics. This perspective could extend into the evaluation of the dFC between specific brain areas, which has the potential to reveal further insights into anomalies of specialized neural populations. Future research should include spatially detailed analyses to better characterize the neural disruptions associated with each disorder. Lastly, to achieve a more complete understanding of fast brain dynamics, additional time-varying measures should be explored to capture different aspects of instantaneous FC in BD and SCZ.

## Conclusion

This study characterizes the fluctuations of dynamic connectivity in patients with BD and SCZ using the aS sequence derived from IAC calculations. Significant differences were found in the mean aS in the delta band for the BD patients and in the gamma band for the SCZ patients. Skewness calculations presented anomalies for both groups in the beta band, extending these alterations into gamma band for the SCZ group. These findings may be linked to widespread and persistent global brain disconnections in BD and an increased prevalence of abnormally high local connectivity values in both BD and SCZ, potentially reflecting pathological neural dynamics.

## Data Availability

The dataset is not publicly available due to restrictions from the Ethics Committee. However, the data supporting the findings of this study are available from S. Iglesias-Parro (siglesia@ujaen.es) upon reasonable request.
